# Serum IL-18 Is a Potential Biomarker for Predicting Severe Dengue Disease Progression

**DOI:** 10.1155/2021/7652569

**Published:** 2021-10-25

**Authors:** Josephine Diony Nanda, Chiau-Jing Jung, Rahmat Dani Satria, Ming-Kai Jhan, Ting-Jing Shen, Po-Chun Tseng, Yung-Ting Wang, Tzong-Shiann Ho, Chiou-Feng Lin

**Affiliations:** ^1^International Ph.D. Program in Cell Therapy and Regenerative Medicine, College of Medicine, Taipei Medical University, Taipei 110, Taiwan; ^2^Department of Microbiology and Immunology, School of Medicine, College of Medicine, Taipei Medical University, Taipei 110, Taiwan; ^3^Graduate Institute of Medical Sciences, College of Medicine, Taipei Medical University, Taipei 110, Taiwan; ^4^International Ph.D. Program in Medicine, College of Medicine, Taipei Medical University, Taipei 110, Taiwan; ^5^Department of Clinical Pathology and Laboratory Medicine, Faculty of Medicine, Public Health and Nursing, Universitas Gadjah Mada, Yogyakarta 55281, Indonesia; ^6^Clinical Laboratory Installation, Dr. Sardjito Central General Hospital, Yogyakarta 55281, Indonesia; ^7^Core Laboratory of Immune Monitoring, Office of Research & Development, Taipei Medical University, Taipei 110, Taiwan; ^8^Department of Pediatrics, National Cheng Kung University Hospital, College of Medicine, National Cheng Kung University, Tainan 704, Taiwan; ^9^Department of Pediatrics, Tainan Hospital, Ministry of Health and Welfare, Tainan 700, Taiwan

## Abstract

*Background*. Dengue virus (DENV) infection is the most common arboviral disease that affects tropical and subtropical regions. Based on the clinical hallmarks, the different severities of patients range from mild dengue fever (MDF) to severe dengue diseases (SDDs) and include dengue hemorrhagic fever or dengue shock syndrome. These are commonly associated with cytokine release syndrome (CRS). The types and levels of cytokines/chemokines, which are suppressed or enhanced, are varied, indicating CRS's pathogenic and host defensive effects. *Principal Finding*. In this study, we created an integrated and precise multiplex panel of cytokine/chemokine assays based on our literature analysis to monitor dengue CRS. A 24-plex panel of cytokines/chemokines was evaluated to measure the plasma levels of targeting factors in dengue patients with an MDF and SDD diagnosis without or with comorbidities. As identified in sixteen kinds of cytokines/chemokines, ten were significantly (*P* < 0.05) (10/16) increased, one was significantly (*P* < 0.01) (1/16) decreased, and five were potentially (5/16) altered in all dengue patients (*n* = 30) in the acute phase of disease onset. Compared to MDF, the levels of IL-8 (CXCL-8) and IL-18 in SDD were markedly (*P* < 0.05) increased, accompanied by positively increased IL-6 and TNF-*α* and decreased IFN-*γ* and RANTES. With comorbidities, SDD significantly (*P* < 0.01) portrayed elevated IL-18 accompanied by increased IL-6 and decreased IFN-*α*2 and IL-12. In addition, decreased platelets were significantly (*P* < 0.05) associated with increased IL-18. *Significance*. These results demonstrate an efficient panel of dengue cytokine/chemokine assays used to explore the possible level of CRS during the acute phase of disease onset; also, we are the first to report the increase of IL-18 in severe dengue with comorbidity compared to severe dengue without comorbidity and mild dengue.

## 1. Introduction

Dengue virus (DENV) is the most prevalent arboviral disease in tropical and subtropical regions [1], which provides the ideal breeding ground for its primary vector, the *Aedes* mosquitoes. It is estimated that 100–400 million people in the world population are infected by DENV infection yearly [2]. DENV infection can be broadly classified according to the degree of severity into mild dengue fever (MDF) and severe dengue diseases (SDDs). The latter can be further distinguished into dengue hemorrhagic fever (DHF) and dengue shock syndrome (DSS), which encompass various symptoms such as circulatory failure or shock, multiorgan failure, and central nervous system (CNS) impairment, which needs intensive treatment and monitoring [3, 4]. This condition exposes patients to a higher risk of death since no effective antiviral treatment is available for dengue infection, and the vaccine has inconsistent effectiveness against the four dengue serotypes [5–7].

Various factors influence the clinical course severity, including virus, host, and environmental factors [8]. The danger of dengue infection also building up in the secondary dengue infection patients as antibodies from the previous infection is being unable to neutralize the virus from heterologous infection, facilitating virus entrance to the Fc-presenting cell, causing higher viral replication and severe infection, known as antibody-dependent enhancement (ADE) [9–13]. In addition, high virus titers can promote overactivation of the immune system and excessive secretion of proinflammatory cytokines, known as cytokine storms or cytokine release syndrome (CRS). This condition may contribute to various symptoms seen in dengue severity [14–16].

Some previous studies have shown that some cytokines are increased in response to severe dengue infection, such as TNF-*α*, MCP-1 (CCL-2), RANTES (CCL-5), IFN-*γ*, IP-10 (CXCL-10), IL-4, IL-6, IL-8 (CXCL-8), IL-10, and GM-CSF (CSF-2) [17–20]. Therefore, changes in the cytokine level might represent some specific event that determines the disease progression. For instance, viral protein nonstructural 1 (NS1) from DENV-infected cells [21] as well as an ADE infectious condition [22] can induce IL-10 production from monocytes. In DENV infection, IL-10 suppresses the DENV-specific T-cell response [23], supporting viral survival and replication and increasing disease severity. On the other hand, a low IFN-*γ* level correlates with a high viral load and more severe symptoms [24], indicating its essential role in early virus elimination and a better disease outcome.

Despite all the previous research, no cytokine marker was approved to predict the severity of dengue. Based on previous observations, a twenty-four-cytokine panel was developed and confirmed using a dengue patient's plasma. We checked their level using a multiplex assay, along with the patient's clinical characteristics. The correlations between severity, clinical characteristics, and cytokine levels were analyzed accordingly.

## 2. Materials and Methods

### 2.1. Article Selection

The articles were retrieved from PubMed (https://pubmed.ncbi.nlm.nih.gov/) in December 2020. The selected publications were published from January 2000 to December 2020. Two keyword combinations of DENGUE AND CYTOKINE AND CLINIC and DENGUE AND CYTOKINE AND ACUTE were used, resulting in 386 and 189 journal papers found, respectively. Both combinations were filtered using “human subjects,” duplicates were discarded, and 384 journals were filtered further using the inclusion and exclusion criteria.

### 2.2. Dengue Disease Severity Classification

Two WHO criteria for DENV infection were used for dengue classification in our literature study. The 2009 criteria classify the severity based on the presence of dengue warning signs. Meanwhile, the older severity classification from 1997 differs between classifications for mild dengue fever (MDF) and severe dengue disease (SDD) through the presence of plasma leakage. The MDF and SDD terms used refer to the previous publication, where the severe dengue disease encompassed dengue hemorrhagic fever (DHF) and dengue shock syndrome (DSS) [25]. Therefore, according to the WHO classification, both WHO severity criteria from 1997 to 2009 are acceptable, as long as the article can define the severity differences of MDF and SDD.

### 2.3. Ethics Statement

All participants gave their informed consent or assent for inclusion before they participated in the study. The study was conducted following the Declaration of Helsinki, and the protocols were approved and carried out following the Institutional Review Boards of National Cheng Kung University Hospital (NCKUH) (reference number A-BR-101-140) and Taipei Medical University (reference number: N201801042), which are organized and operated according to the laws and regulations of Good Clinical Practice (ICH-GCP).

### 2.4. Blood Sample

Thirty patients, including MDF patients (*n* = 10) and SDD patients without (*n* = 10) or with (*n* = 10) comorbidities, admitted to the NCKUH were enrolled in the study. All enrolled patients were classified into three groups according to 2009 WHO criteria based on levels of severity: (1) group A: dengue without warning signs; (2) group B: dengue with warning signs (abdominal pain, persistent vomiting, fluid accumulation, mucosal bleeding, lethargy, liver enlargement, and increasing hematocrit with decreasing platelets); and (3) group C: severe dengue (dengue with severe plasma leakage, severe bleeding, or organ failure [Reference: WHO, WHO Guidelines Approved by the Guidelines Review Committee, in Dengue: Guidelines for Diagnosis, Treatment, Prevention and Control: New Edition. 2009, World Health Organization]).

Primary demographic data, medical history, physical examination data, and subsequent progress were recorded on a standard data form for each patient. For dengue patients, blood was taken before 6 days for the acute febrile (*n* = 30) and in between 9 and 18 days for the convalescent phase (*n* = 30) from the disease onset, as defined according to the guidelines of the CDC (https://www.cdc.gov/dengue/healthcare-providers/clinical-presentation.html). In addition, six healthy individuals were also enrolled to collect blood samples as controls. All six participants were confirmed to be healthy volunteers who were not taking any medication and were free from recent infectious diseases. Each participant took ten milliliters of blood using sodium heparin BD vacutainer collection tubes (5 mL; Becton Drive Vacutainer, Franklin Lakes, USA). Plasma was separated and prepared using a conventional method as described elsewhere (https://www.thermofisher.com/tw/zt/home/references/protocols/cell-and-tissue-analysis/elisa-protocol/elisa-sample-preparation-protocols/plasma-and-serum-preparation.html), and plasma samples were stored at -80°C until use.

### 2.5. Clinical Blood Parameters

Patients' clinical blood parameters were tested using the standard protocols in the Department of Pathology, NCKUH. Total circulating white blood cell counts (WBCs), platelet counts, and hematocrit were obtained by the standard complete blood count test. In addition, a DENV diagnosis was confirmed using one or more examinations: positive for plasma nonstructural protein 1 (NS1) antigen, dengue IgM antibodies detected using a kit (Bioline Dengue Duo™; Standard Diagnostics, Seoul, Korea), or DENV RNA detected using real-time reverse transcriptase-polymerase chain reaction (RT-PCR) (TIB Molbiol, Lightmix kit; Roche Applied Science, Berlin, Germany) [26]. For viral NS1 measurement, NS1 ELISA was performed using paired anti-NS1 antibodies that were prepared in our laboratory, and the ELISA was quantified by the addition of 100 *μ*L of 3,3′,5,5′-tetramethylbenzidine (TMB) substrate (R&D Systems) [27].

### 2.6. Cytokine/Chemokine Measurement

The cytokines investigated were IL-10, IFN-*γ*, IL-6, TNF-*α*, IL-8 (CXCL-8), CXCL-10 (IP10), CCL-2 (MCP-1), IL-4, CCL-4 (MIP-1*β*), IFN-*α*, CCL-5 (RANTES), GM-CSF (CSF-2), IL-15, VEGF (VEGFA), IL-13, IL-18, IL-1R*α*, IL-12 (IL-12p70), CCL-3 (MIP-1*α*), CXCL-9 (MIG), IL-17 (IL-17A), IL-1*β*, IL-7, and IL-2, as noted in Table 1. Undiluted plasma cytokine/chemokine concentrations were detected using the integrated Milliplex Human Cytokine MAGNETIC BEAD 24 Plex customized kit per the manufacturer's instructions (https://www.merckmillipore.com/TW/zh/product/MILLIPLEX-MAP-Human-Cytokine-Chemokine-Magnetic-Bead-Panel-Immunology-Multiplex-Assay, MM_NF-HCYTOMAG-60K#anchor_TI). Raw data were collected as relative fluorescence intensities and converted to cytokine concentrations in pg/mL using the standard curve generated from the reference concentrations. The concentration below the limit of detection was assumed to be equal to 0 pg/mL.

### 2.7. Statistical Analysis

All data outliers were sorted and excluded from the analysis. In the heat map, the cytokine value was normalized to each cytokine level. Correlations between demographic data (age, hospital time, and ICU duration) and severity, clinical parameters, and cytokines were tested using Spearman's correlation. Meanwhile, Fisher's exact test was used to evaluate sex and serology characteristic correlations with the severity (MDF, SDD-C, and SDD+C). Dengue infection comorbidity was analyzed using biserial point correlation. The Mann-Whitney test was used to analyze group differences in cytokine levels in healthy versus dengue, MDF versus SDD, and SDD-C versus SDD+C. The Wilcoxon matched-pair signed-rank test was used in the clinical characteristics for the acute versus convalescent phase. For more than two groups (WBC, hematocrit, platelet, and NS1 OD in three different severity groups and two phases), the Kruskal-Wallis test was used. The significant result was further examined for the *post hoc* test. Both tests were investigated in a two-sided test with a 95% confidence interval (95% CI). These analyses were performed using IBM SPSS Statistics (version 19.0.0) and GraphPad Prism (version 8.3.0), and graphs were generated from GraphPad Prism (version 8.3.0). Statistical significance was set at *P* < 0.05.

## 3. Results

### 3.1. Design of an Integrated and Precise Multiplex Cytokine/Chemokine Panel Based on a Literature Study

**Figure 1:**
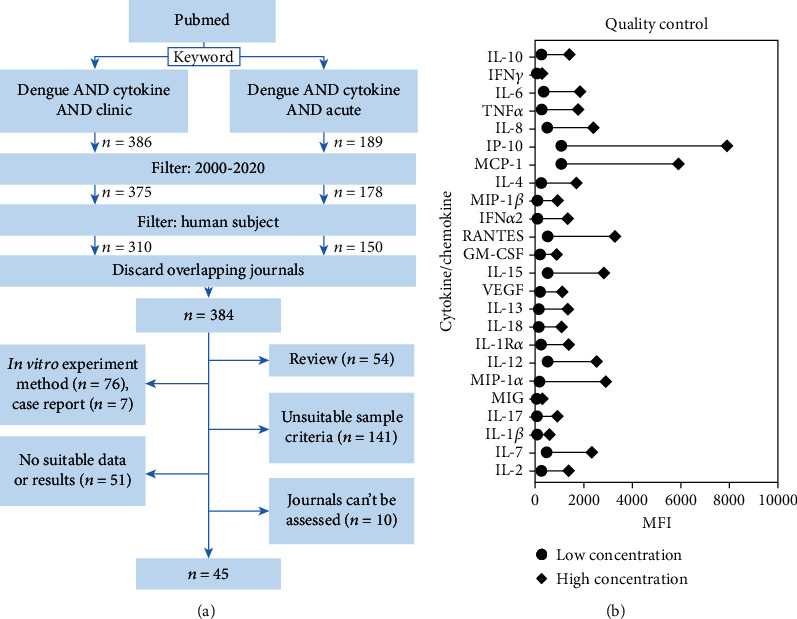
Cytokine/chemokine panel development flow and multiplex quality control. (a) Flow diagram of article selection progress for making the integrated and precise dengue cytokine/chemokine panel. Articles were taken from PubMed and filtered using the inclusion and exclusion criteria, which yielded 45 articles used in the analysis. (b) Quality control of the multiplex assay presented as the mean fluorescent intensity (MFI) for each cytokine/chemokine measured at low and high concentrations.

To design a precise multiplex cytokine/chemokine panel for investigating CRS in dengue patients, a literature study according to previous works was conducted and summarized in Figure 1(a). Three hundred eighty-four articles were retrieved from PubMed and were further filtered using the inclusion and exclusion criteria. The inclusion criteria were as follows: (1) clinical study using adult dengue patient samples: blood or plasma (systemic indication), (2) acute phase/febrile phase/before the 7th day from disease onset, and (3) an original article. The exclusion criteria were as follows: (1) *in vitro* or *in vivo* study, (2) nondengue-related infection or other infection or coinfection, (3) subject treated or vaccinated, (4) subject below 17 years old, and (5) results that did not provide significance in DHF cytokine to MDF/healthy or MDF to healthy. The filtration resulted in the exclusion of 339 studies through a quick review of the abstracts or the full text due to being a review (*n* = 54) and an *in vitro* experiment method (*n* = 76), a case report (*n* = 7), having an unsuitable sample criteria: not human (*n* = 20), child (*n* = 35), not using serum/plasma (*n* = 14), having other infections or coinfections (*n* = 25), getting treatment or a vaccine (*n* = 34), having no suitable time point (*n* = 1), having no suitable data or result (*n* = 51), the journal was not available (*n* = 7), the article was not reported in English (*n* = 2), and a retracted article (*n* = 1), giving the final result of 45 papers to be evaluated.

Accordingly, an integrated multiplex panel of cytokines/chemokines was constructed (Table 1) by comparing the frequency of reported matched cytokines and chemokines in severe dengue and less severe cases adapted from the results of the 45 papers. As a result, a significant increase is listed from each article to obtain the top 24 cytokines from each included article (Table S1). Based on the results, the top 6 kinds of cytokines/chemokines reported in over 10 papers were IL-10, IFN-*γ*, IL-6, TNF-*α*, IL-8 (CXCL-8), and IP-10 (CXCL-10). The others included MCP-1 (CCL-2), IL-4, MIP-1*β* (CCL-4), IFN-*α*2, RANTES (CCL-5), GM-CSF (CSF-2), IL-15, VEGF (VEGFA), IL-13, IL-18, IL-1R*α*, IL-12 (IL-12p70), MIP-1*α* (CCL-3), MIG (CXCL-9), IL-17 (IL-17A), IL-1*β*, IL-7, and IL-2.

To analyze multiple cytokine and chemokine biomarkers in human plasma simultaneously, we used bead-based multiplex assays using commercial and customized assays, as summarized in Table 1. Quality controls (QCs) are included in these specialized panels to control and verify the assay performance to assure pipetting quality and the assay setup. Based on the results of Luminex technology measurement, the QC conferred two range concentrations of cytokine/chemokine analyses, which included high and low concentrations (Figure 1(b)).

### 3.2. Dengue Patient Demographic and Clinical Characteristics

The number of dengue patients who were diagnosed using the standard procedure (please refer to Materials and Methods) involved in this study was 30, and the patients were divided into three severity groups: mild dengue fever (MDF, *n* = 10), severe dengue disease (SDD) without comorbidities (SDD-C) (*n* = 10), and SDD with comorbidities (SDD+C) (*n* = 10). As summarized in Table 2, all the patients (17 male and 13 female) had a mean age of 61.6 years old, and age was weakly correlated with severity (*P* = 0.01). The mean hospital time and ICU duration were 0 days in the mild group, 10 and 3 days in the SDD-C group, and 29.4 and 9.1 days in the SDD+C group. These two variables strongly correlated with severity (*P* < 0.001), indicating the importance of medical care in severe dengue infection. The common comorbidities, which also had a strong correlation with severity, found in the patients were diabetes mellitus (DM) and hypertension (HT) (*P* < 0.001), with 1 and 2 patients in the MDF group and 8 and 10 patients in the SDD+C group, respectively. The other comorbidities, chronic renal failure (CRF), cardiovascular accidents (CVA), and chronic obstructive pulmonary disease (COPD), only had one patient each in the SDD+C group and thus had no significant correlations with the severity.

Serologic markers were not tested on all dengue patients. In the MDF and SDD-C groups, only 7 patients were tested, but 9 were tested in the SDD+C group. Two patients were positive for IgM in the MDF group. In both the SDD-C and SDD+C groups, there were 3 IgM-positive patients in each group. Furthermore, we checked whether the day that the sample was taken influenced the presence of IgM. Our results showed that, on average, IgM-positive patient samples were taken on day 3.2 after fever onset. However, several other patients with negative results had samples taken in a similar time range. Meanwhile, dengue IgG was detected in an MDF patient group and 2 and 4 in the SDD-C and SDD+C groups, respectively. This antibody might be used as a marker for secondary dengue infection, demonstrating the function and memory of the immune system. NS1 was positive in most patients that were checked in all the severity groups. Six of seven patients in MDF, all patients in SDD-C, and 8 of 9 in the SDD+C group had NS1 levels detected.

Clinical parameters checked in a quantitative assay were white blood cell (WBC) counts, hematocrit, platelet counts, and NS1. In two phases (acute and convalescent), WBC was the highest in the SDD+C group. In the acute phase, SDD-C has a lower concentration than the MDF group. Meanwhile, MDF had the lowest count in the convalescent phase, followed by the SDD-C group (Figure S1A). The hematocrit percentage was slightly different between each severity group in the acute and convalescent phases (Figure S1B), suggesting that severity does not influence the hematocrit changes in the dengue patients tested in this study. For the platelet count, a significant difference was observed in the overall acute dengue compared to convalescent dengue (*P* < 0.01) (Table S2). The platelet level peaked in the acute phase of the MDF group and decreased as the disease progressed to severe (Table S3). The platelet count pattern in the acute phase was also reflected in the convalescent phase, where the increased level of platelets in the severe group exceeded that in the MDF group (Figure S1C). In contrast to platelets, the NS1 level was the lowest in the MDF group of the acute phase and at its peak in the SDD-C group, slightly higher than that in the SDD+C group (Figure S1D). These levels decreased to near zero in the convalescent phase, resembling each phase's virus replication condition. We also checked the correlation between four clinical indicators, including WBC, platelet, hematocrit, and NS1. Only the platelets and the NS1 levels had a significant negative correlation (*P* < 0.01) (Figure S2). Moreover, patients with SDD generally showed severe thrombocytopenia accompanied by increased NS1 toxemia at the acute phase of disease onset.

### 3.3. The General Pattern of Cytokine/Chemokine Levels in Healthy Individuals with MDF, SDD-C, and SDD+C

**Figure 2:**
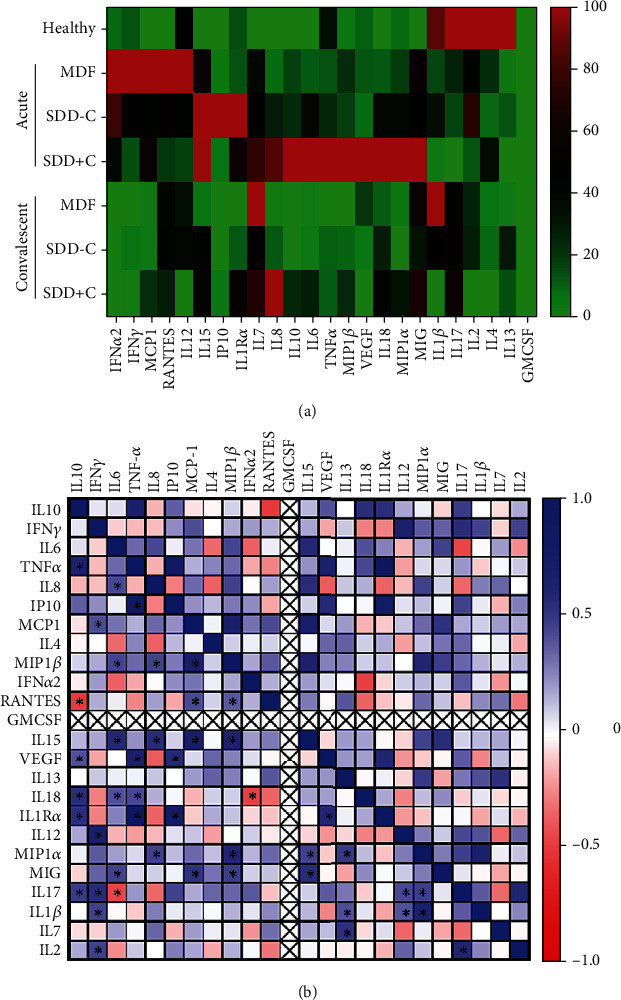
Cytokine/chemokine concentrations in healthy and dengue patients. (a) The cytokine/chemokine levels in healthy and dengue patients were presented in the heat map. The concentration of each cytokine was normalized and presented as a percentage compared to the other group for the same cytokine. MDF: mild dengue fever; SDD-C: severe dengue disease without comorbidities; SDD+C: severe dengue disease with comorbidities. (b) The heat map shows a correlation study between each cytokine/chemokine. Significant values are marked with a star (∗) inside the area. The blue color indicates a positive correlation, and the red color indicates a negative correlation. The GM-CSF value cannot be assessed because its level is below the detection range.

To depict the pattern of cytokine/chemokine levels in the acute febrile phase of dengue patients, we measured and mapped the cytokine/chemokine levels in healthy and dengue patients. A group of interferon (IFN) cytokines (IFN-*α*, IFN-*β*, and IFN-*γ*) is known to play a role in protecting cells from DENV infection *in vitro* [28] and inhibiting DENV replication *in vivo* [29]. This condition reflects our finding (Figure 2(a)), where the IFN cytokines (IFN-*α*2 and IFN-*γ*) and antivirus-associated IL-12, MCP-1, and RANTES in the acute febrile phase of dengue patients were higher than those in healthy controls, as they play a role in defending against DENV infection. However, their level in the acute SDD group was lower than that in the MDF group, indicating an immune response inability to defend against the infection, thus promoting the progression to severe infection. The IFN levels in the convalescent phase are similar to those in healthy individuals since the pathogens are already cleared out. Another interesting finding was that multiple cytokines, including IL-7, IL-8, IL-6, IL-10, IL-18, MIG, MIP-1*α*, MIP-1*β*, TNF-*α*, and VEGF, increased prominently in the acute SDD+C group compared to the other groups. These cytokines might be involved in dengue CRS and may be potential markers for severity in dengue patients.

Cytokine level correlations between various cytokines in the acute phase of DENV infection were evaluated and plotted in a heat map. Significant correlations between cytokine pairs are marked with an asterisk sign. As shown in the heat map, IL-10 is positively associated with TNF-*α*, VEGF, IL-18, IL-1R*α*, and IL-17 and negatively with RANTES. IFN-*γ* was positively correlated with MCP-1, IL-12, IL-17, IL-1*β*, and IL-2. IL-6 was positively linked with IL-8, MIP-1*β*, IL-15, IL-18, and MIG, but negatively with IL-17. IP-10, VEGF, IL-18, and IL-1R*α* were positively correlated with TNF-*α*. IL-8 was directly correlated with MIP-1*β*, IL-15, and MIP-1*α*. Both VEGF and IL-1R*α* have a positive relationship with IP-10. MCP-1 positively correlates with MIP-1*β*, RANTES, IL-15, and MIG. A positive association was also found between MIP-1*β* and RANTES, IL-15, MIP-1*α*, and MIG. On the other hand, IFN-*α*2 is linked with IL-18. IL-15 has a positive correlation with MIP-1*α* and MIG. VEGF was positively associated with IL-1R*α*. IL-13 was directly correlated with MIP-1*α*, IL-1*β*, and IL-7. Both IL-12 and MIP-1*α* were correlated with IL-17 and IL-1*β*. IL-17 was positively correlated with IL-2. Furthermore, we defined a correlation with a coefficient higher than 0.7 as vital and found 3 potent linear correlation cytokines, duo, IP-10 to TNF-*α*, together with IL-1*α* to TNF-*α* and IP-10 (Figure 2(b)).

### 3.4. High and Low Cytokine/Chemokine Patterns in Acute Dengue Patients Compared to Healthy Controls

**Figure 3:**
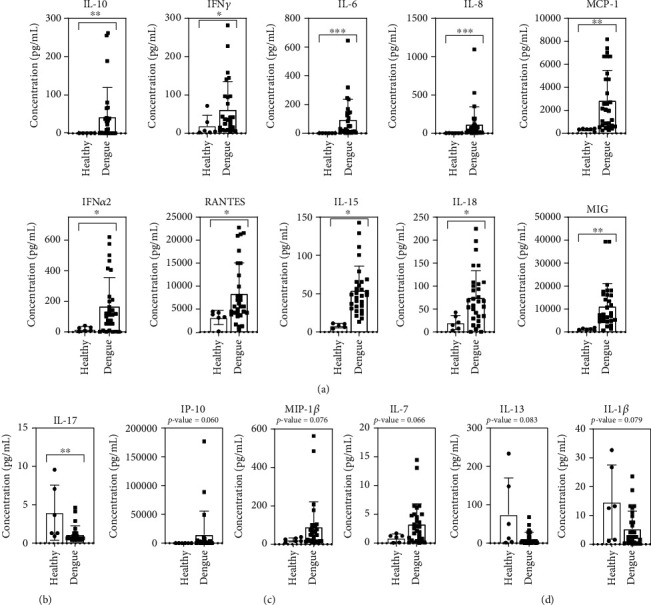
Cytokines/chemokines were significantly different between healthy and dengue patients. The acute phase dengue group had cytokine/chemokine levels of (a) IL-10, IFN-*γ*, IL-6, IL-8, MCP-1, IFN-*α*2, RANTES, IL-15, IL-18, and MIG that were significantly higher and of (b) IL-17 that were dramatically lower than healthy controls. (c) Dengue patients had higher IP-10, MIP-1*β*, and IL-7 than in healthy controls. (d) The cytokine/chemokine levels higher in healthy patients than in dengue patients were IL-13 and IL-1*β*. The comparison was done using Mann-Whitney test. ^∗^*P* < 0.05, ^∗∗^*P* < 0.01, and ^∗∗∗^*P* < 0.001.

We analyzed the mean difference in cytokine/chemokine expression in the healthy group (*n* = 6) compared to all acute febrile phases of DENV infection (*n* = 30) (Table S4). The results showed a significant (*P* < 0.05) increase in immune factors at the acute febrile phase of disease onset, including in 10 of 24 cytokines/chemokines (IL-10, IFN-*γ*, IL-6, IL-8, MCP-1, IFN-*α*2, RANTES, IL-15, IL-18, and MIG). The top three cytokines with the highest significant differences (all *P* ≤ 0.001) were IL-6, IL-8, and IL-15 (Figure 3(a)). One cytokine level, the IL-17 level, was significantly lower in acute dengue patients than in healthy controls (*P* = 0.007) (Figure 3(b)). Other cytokines/chemokines that showed a borderline significant increase in acute dengue compared with healthy ones were IP-10, MIP-1*β*, and IL-7 (Figure 3(c)). On the other hand, IL-13 and IL-1*β* values were lower in acute dengue patients (Figure 3(d)). When we compared this result to the convalescent phase, most of these factors were decreased, except IL-17 and IL-7 (Table S4). The increased cytokines/chemokines are speculated to be pathogenic and protective in response to DENV infection.

### 3.5. The Levels of IL-8 and IL-18 Were Significantly Higher in the SDD Groups than in the Acute MDF Groups

**Figure 4:**
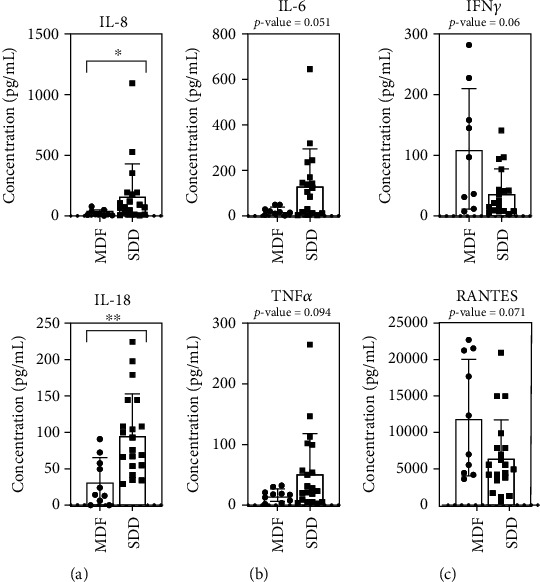
Cytokines/chemokines with significant differences in MDF and SDD patients. (a) The group in which cytokine/chemokine levels are significantly higher in SDD than MDF was IL-8 and IL-18. (b) Meanwhile, IL-6 and TNF-*α* are near significant. (c) The group in which cytokine/chemokine levels are nearly higher in MDF than in SDD was seen in IFN-*γ* and RANTES. The comparison was done using Mann-Whitney Test. ^∗^*P* < 0.05 and ^∗∗^*P* < 0.01.

According to the WHO classification, we further divided the dengue patients into MDF (*n* = 10) and SDD (*n* = 20) and compared their cytokine/chemokine expression levels. Our findings showed the varied expression of cytokines/chemokines in acute SDD and MDF. It was shown especially in IL-8 and IL-18, where SDD cytokine/chemokine levels were significantly higher in SDD than MDF (IL-8, *P* = 0.037, and IL-18, *P* = 0.002) (Table S5 and Figure 4(a)). A similar borderline-significant pattern was also observed for IL-6 and TNF-*α*, with a higher expression in the SDD group than in the MDF group (IL-6, *P* = 0.051, and TNF-*α*, *P* = 0.094) (Table S5 and Figure 4(b)). In contrast, based on the results (Table S5 and Figure 4(c)), the cytokine levels showed a decreasing trend that was not statistically significant (IFN-*γ*, *P* = 0.06, and RANTES, *P* = 0.071) in the acute phase of the SDD group (IFN-*γ*, $\overset{®}{x}=38.4\text{ pg}/\text{mL}$, and RANTES, $\overset{®}{x}=6515.1\text{ pg}/\text{mL}$) compared to the MDF group (IFN-*γ*, $\overset{®}{x}=110.6\text{ pg}/\text{mL}$, and RANTES, $\overset{®}{x}=12021.6\text{ pg}/\text{mL}$), indicating an inability of the immune response to defend against the infection, thus promoting the progression to severe infection. Therefore, increased IL-18 and IL-8, probable increased IL-6 and TNF-*α* together with decreased IFN-*γ* and RANTES in acute SDD patients resemble cytokine involvement in severe dengue progression.

### 3.6. The Increased Cytokine/Chemokine Pattern in SDD with Comorbidities

**Figure 5:**
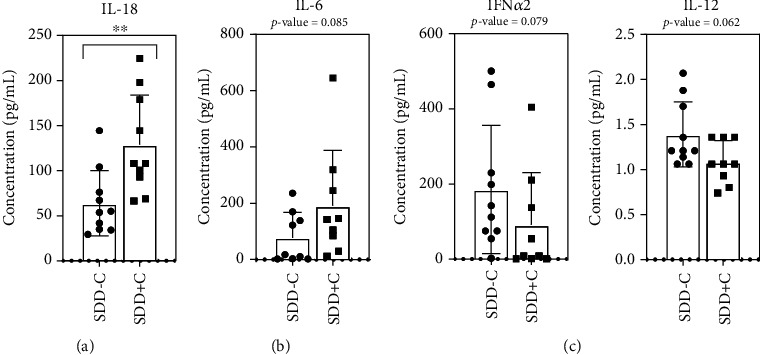
Cytokines/chemokines with significant differences in the two groups of severe dengue patients, SDD-C and SDD+C. The cytokine/chemokine levels of IL-18 (significant) (a) and IL-6 (near significant) (b) were higher in the SDD+C group than in the SDD-C group. (c) The groups in which cytokine/chemokine levels were nearly as high in SDD-C as in SDD+C which were IFN-*α*2 and IL-12. The comparison was done using Mann-Whitney test. ^∗∗^*P* < 0.01.

Afterward, we compared the cytokine/chemokine levels in severe dengue patients with and without comorbidities (SDD-C and SDD+C), primarily at the acute febrile phase of disease onset. Several cytokines/chemokines showed an increasing trend over twofold in SDD+C compared with SDD-C, such as IL-10, IL-6, TNF-*α*, IL-8, IL-4, MIP-1*β*, VEGF, and IL-18 (Table S6), with the most significant changes found in IL-18 (*P* < 0.01) (Figure 5(a)). A significant-likelihood pattern similar to IL-18 was also observed in IL-6 (*P* = 0.085) (Figure 5(b)). In contrast, there was nonsignificance over the twofold decreasing trend in SDD+C compared with SDD-C, such as IFN-*γ*, IP-10, IFN-*α*2, IL-13, IL-1*β*, and IL-2 (Table S6), with the near-significant fewer production of IFN-*α*2 (*P* = 0.079) and IL-12 (*P* = 0.062) (Figure 5(c)). Thus, increased IL-18 and other altered cytokines/chemokines are associated with the development of comorbidities in SDD patients.

### 3.7. Correlation between Clinical Parameters and Cytokine/Chemokine Expression

**Figure 6:**
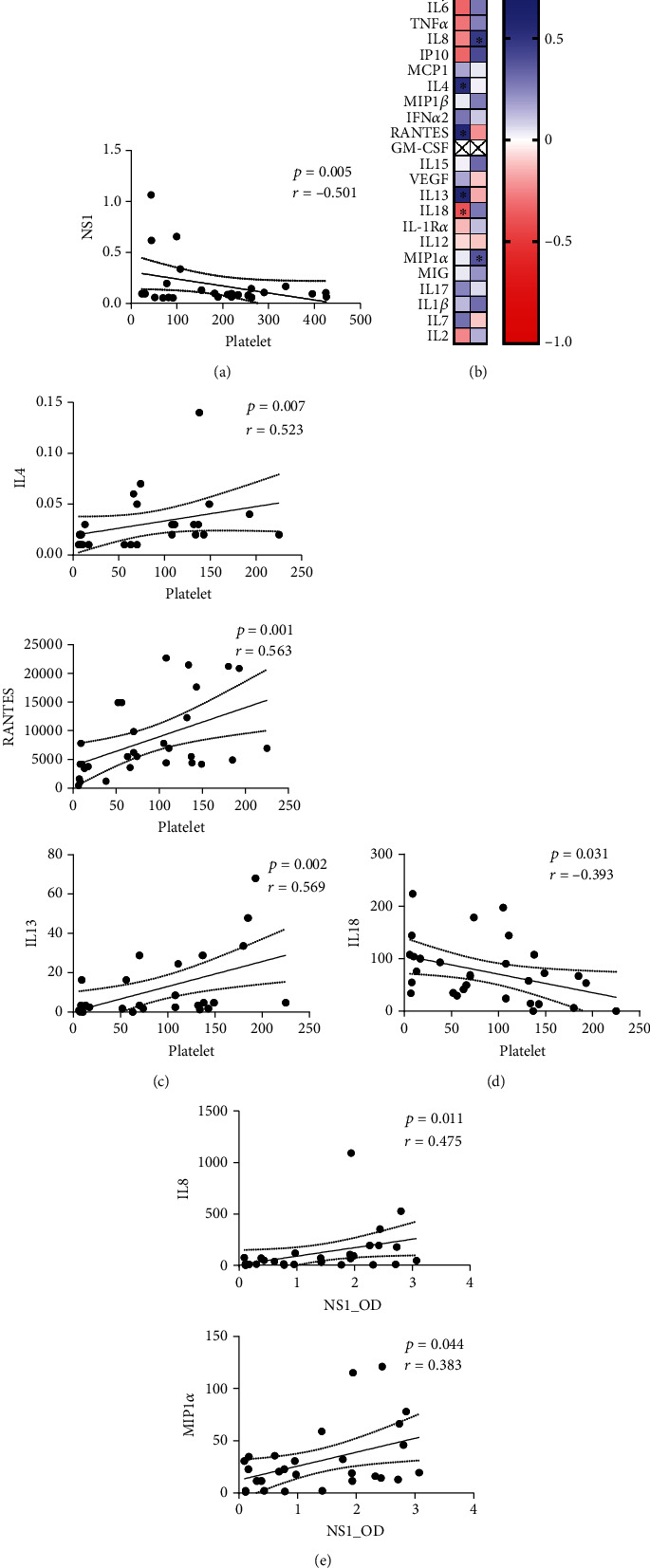
Correlation between clinical parameters and cytokines. The correlation showed a marked negative correlation between NS1 and platelets (a) and its levels to other cytokine/chemokine groups (b). Significant values are marked with a star (∗) inside the area. The blue color indicates a positive correlation, and the red color indicates a negative correlation. A significant correlation was found in platelets positively with IL-4, IL-13, and RANTES (c) but negatively with IL-18 (d). (e) NS1 was positively correlated with IL-8 and MIP-1*α*. The correlation was performed using the Spearman correlation test. A significant change was set on the *P* value < 0.05.

While checking the correlation between four clinical indicators (WBC, hematocrit, platelet, and NS1), we found that NS1 and platelet counts were negatively associated (*r* = −0.501, *P* = 0.005) (Figure S2 and Figure 6(a)). Based on the findings, a correlation between NS1 and platelets with the tested 24 cytokine/chemokine levels was next assessed (Figure 6(b)). We found a significant direct correlation between platelets and IL-4 (*r* = 0.523, *P* = 0.007), RANTES (*r* = 0.563, *P* = 0.001), and IL-13 (*r* = 0.569, *P* = 0.002) (Figure 6(c)), with a significant negative correlation with IL-18 (*r* = 0.393, *P* = 0.031) (Figure 6(d)). Meanwhile, NS1 had a marked positive relationship with IL-8 (*r* = 0.475, *P* = 0.011) and MIP-1*α* (*r* = 0.383, *P* = 0.044) (Figure 6(e)). The correlations between platelets, NS1, and possible cytokines/chemokines could represent the interaction involved in the progression of dengue diseases and immunomodulation in response to DENV infection.

## 4. Discussion

Multiple factors affect the incidence of severe dengue disease progression, not only the virus strain, vector control, and environmental deviation [30] but also aging [31] and comorbidities [32]. We examined dengue patients' clinical and cytokine/chemokine profiles using blood samples in the present study. Our study is consistent with previous works [31, 33], and our study also validated that SDD is associated with aging and chronic underlying diseases, such as DM and hypertension. Older people and people with comorbidities are at-risk populations for developing severe diseases in response to DENV infection [31]. In addition to host factors, serological and hematological parameters conferred that in SDD patients, the NSI levels were increased, and on the other hand, circulating platelet levels were decreased. Our current work also provides unique cytokine/chemokine biomarkers for predicting the severity of dengue diseases.

By using the integrated and precise 24-plex cytokine/chemokine panel, our findings demonstrated an increase in ten (IFN-*α*2, IFN-*γ*, IL-6, IL-8, IL-10, IL-15, IL-18, IP-10, MCP-1, MIG, and RANTES) and a decrease in one (IL-17A) cytokine/chemokine in all dengue cases compared with healthy subjects. Among these factors, increases in IFN-*γ* and IL-10 are consistent with previous studies [34]. Similar to the previous study [35], our finding showed a significant increase in IL-8 and IL-18, with an immediate increase in IL-6 and TNF-*α* in SDD to MDF patients. However, TNF-*α* trends showed a reversed pattern with our findings, where MDF has a higher level than DHF [36]. On the other hand, reduction of IFN-*γ* and RANTES levels in SDD resembles previous findings showing IFN-*γ* production suppression in DHF or DSS [37]. A study in mice further enhanced the IFN-*γ* role in preventing the systematic replication of DENV and spread to the CNS [38].

Notably, we compared cytokine/chemokine levels in SDD without (SDD-C) and with comorbidity (SDD+C), with the most prevalent being DM and hypertension. Between these two groups, IL-18 was increased significantly in the SDD+C to the SDD-C group, with the borderline significant increase of IL-6 and decrease of IFN-*α*2 and IL-12. A similar result has not been reported before, even though DM and hypertension are already widely known to increase the risk of severe progression in dengue patients [19, 33, 39]. However, the possible effects of DENV infection on comorbid host susceptibility and the involvement of existing comorbidities in facilitating host immune overactivation in response to DENV infection and disease progression are undocumented.

Our results represent that IL-18 is the cytokine that steadily increased significantly in all the severity groups and was shown to be severe in the comorbid group (Figure S3). Even though various reports about diabetes and hypertension influencing dengue severity have been reported before [33, 40, 41], we are the first to report the significant increase of the IL-18 level in dengue-infected patients with comorbidities to dengue-infected patients without comorbidities. IL-18 is a cytokine secreted from activated monocytes/macrophages and can induce IFN-*γ* secretion in severe dengue [42]. A previous report showed a similar result, where IL-18 significantly increases in severe dengue compared to the healthy samples of patients with mild dengue infection [43, 44]. Although the possible role of IL-18 in facilitating dengue disease progression remains undefined, altered IL-18 production has been reported in various inflammatory conditions, for example, in sepsis [45], diabetes [46], metabolic syndromes [47, 48], atherosclerosis [49, 50], and cardiovascular disorders [50].

Interestingly, we also find a significant negative correlation between platelet to NS1 and IL-18 levels in the acute phase of the dengue infection. One reason for this association could be since NS1 alone is sufficient to induce platelet activation in dengue infection [51], while platelet contains the IL-18 gene that will be transcripted when activated [52]. Thus, significant platelet induction will cause a considerable increase in the serum IL-18. Platelet activation will eventually lead to aggregation, adherence to endothelium, and trigger phagocytosis by macrophage [51], mediated by scavenger receptor type A in the macrophage. Further, the interaction between autologous activated platelets and macrophages will promote proinflammatory cytokine production [53]. Besides the dengue infection itself, the macrophage cytokine also facilitates higher spleen B cell IgM and IgG production in the presence of dengue virus, which is believed to be responsible for dengue clearance [54, 55]. In the long run, progressive reduction of IgG concentration might expose the host risk to ADE in the heterologous dengue infection when its level is below the minimum neutralization requirement. ADE will induce more DENV infection and replication inside Fc-*γ*-receptor- (Fc*γ*R-) expressing cells and promote more proinflammatory cytokine secretion, including IL-18 [56, 57].

Our research does have certain limitations. Although gender differences in dengue severity have been described earlier, this study did not evaluate gender due to sample size limitations. The small sample size may have hampered the study's precision. However, the detailed mechanism by which IL-18 might contribute to the disease severity, by either exacerbating or preventing it, is still unclear. This can be investigated further in future studies. Finally, we discovered that IL-18 levels were significantly higher in the various group circumstances, making it a plausible option for a dengue severity measure, particularly when severe with comorbid groups.

## Figures and Tables

**Table 1:** Literature study of increased plasma/serum levels of cytokine/chemokine expression in adult dengue patients and design of premixed panel of cytokine/chemokine as dengue cytokine 24-plex panel.

Number	Cytokine/chemokine	Reported (time)	Catalog
1	IL-10	21	HIL10-MG
2	IFN-*γ*	19	HIFNG-MG
3	IL-6	15	HIL6-MG
4	TNF-*α*	15	HTNFA-MG
5	IL-8 (CXCL-8)	13	HIL8-MG
6	IP-10 (CXCL-10)	12	HIP10-MG
7	MCP-1 (CCL-2)	9	HMCP1-MG
8	IL-4	7	HIL4-MG
9	MIP-1*β* (CCL-4)	7	HMIP1B-MG
10	IFN-*α*	7	HIFNA2-MG
11	RANTES (CCL-5)	6	HRANTES-MG
12	GM-CSF (CSF-2)	6	HGMCSF-MG
13	IL-15	6	HIL15-MG
14	VEGF (VEGFA)	4	HVEGFA-MG
15	IL-13	4	HIL13-MG
16	IL-18	4	HIL18-MG
17	IL-1R*α*	4	HIL1RA-MG
18	IL-12 (IL-12p70)	3	HIL12P70-MG
19	MIP-1*α* (CCL-3)	3	HMIP1A-MG
20	MIG (CXCL-9)	3	HMIG-MG
21	IL-17 (IL-17A)	3	HIL17A-MG
22	IL-1*β*	3	HIL1B-MG
23	IL-7	2	HIL7-MG
24	IL-2	2	HIL2-MG

**(a):** 

Demographic characteristic	MDF (*n* = 10)	SDD-C (*n* = 10)	SDD+C (*n* = 10)	Total (*n* = 30)	*P* value	Correlation coefficient
Age (mean ± SD, y.o.)	47.8 ± 23	62.3 ± 20.4	74.8 ± 6.3	61.6 ± 20.8	**0.010**	0.465
Gender (*n*)						
Male	6	3	8	17	0.103	—
Female	4	7	2	13		
Hospital time (mean, days)	0	10	29.4	13.1	**<0.0001**	0.883
ICU duration (mean, days)	0	3	9.1	3.9	**<0.0001**	0.713
Comorbid (*n*)						
Diabetes mellitus	1	0	8	9	**<0.001**	0.624
Hypertension	2	0	10	12	**<0.001**	0.667
CRF	0	0	1	1	1	—
CVA	0	0	1	1	1	—
COPD	0	0	1	1	1	—

**(b):** 

Serology characteristic	MDF (*n* = 7)	SDD-C (*n* = 7)	SDD+C (*n* = 9)	Total (*n* = 23)	*P* value	Correlation coefficient
Dengue IgM (*n*)						
Positive	2	3	3	8	1	—
Negative	5	4	6	15		
Dengue IgG (*n*)						
Positive	1	2	4	7	0.465	—
Negative	6	5	5	16		
NS1 (*n*)						
Positive	6	7	8	21	0.739	—
Negative	1	0	1	2		

The data represent demographic data of the dengue patients used in this research. Age has a weak correlation with severity; meanwhile, hospital time, ICU duration, DM, and hypertension strongly correlate with severity. There was no correlation between NS1, IgG, and IgM and the severity of dengue in patients. Age, hospital time, and ICU duration were analyzed using the Spearman correlation. Sex and serology characteristics were analyzed separately using Fisher's exact test. Comorbid in dengue infection analyzed using point biserial correlation. Bold indicates a significant *P* value. A significant change was set on the *P* value < 0.05. MDF: mild dengue fever; SDD-C: severe dengue disease without comorbidity; SDD+C: severe dengue disease with comorbidities; DM: diabetes mellitus; CRF: chronic renal failure; CVA: cardiovascular accidents; COPD: chronic obstructive pulmonary disease; Ig: immunoglobulin; NS1: nonstructural protein 1.

## Data Availability

The data used to support the findings of this study are available from the corresponding authors upon request.

## Supplementary Materials

Supplementary Materials S1 Table: literature study of increased plasma/serum levels of cytokine/chemokine expression in adult dengue patients. S2 Table: clinical characteristics in dengue patients. S3 Table: clinical hematological characteristics in dengue patients. S4 Table: healthy, acute, and convalescent dengue cytokine/chemokine level. S5 Table: MDF and SDD cytokine/chemokine concentration in the acute and convalescent phase of disease onset. S6 Table: cytokine/chemokine concentration in acute SDD-C versus SDD+C patients. S1 Figure: clinical characteristics in dengue patients. Clinical parameters, including (A) White blood cells, (B) hematocrit, (C) platelet, and (D) NS1, were shown. Data were analyzed using the Kruskal-Wallis test. ^∗^*P* < 0.05, ^∗∗^*P* < 0.01, and ^∗∗∗^*P* < 0.001. S2 Figure: correlation test between clinical characteristics in acute dengue patients. The heat map showed a correlation study between platelet, NS1, WBC, and hematocrit. Significant value marked with a star (∗) inside the area. The blue color indicates a positive correlation, and the red color indicates a negative correlation. WBC: white blood cells; NS1: nonstructural protein 1; OD: optical density. Data were analyzed using the Spearman comparison test. ^∗∗^*P* < 0.01. S3 Figure: IL-18 level in healthy, MDF, SDD-C, and SDD+C. The graph showed the difference between groups in IL-18 concentration. Data were analyzed using the Kruskal-Wallis test. ^∗∗^*P* < 0.01 and ^∗∗∗^*P* < 0.001.
