# Supplementary material for: Serum IL-18 Is a Potential Biomarker for Predicting Severe Dengue Disease Progression
Source: J Immunol Res. 2021 Oct 25;2021:7652569. doi: 10.1155/2021/7652569 (PMC8560270; doi:10.1155/2021/7652569)
Supplement: Supplementary Materials — S1 Table: literature study of increased plasma/serum levels of cytokine/chemokine expression in adult dengue patients. S2 Table: clinical characteristics in dengue patients. S3 Table: clinical hematological characteristics in dengue patients. S4 Table: healthy, acute, and convalescent dengue cytokine/chemokine level. S5 Table: MDF and SDD cytokine/chemokine concentration in the acute and convalescent phase of disease onset. S6 Table: cytokine/chemokine concentration in acute SDD-C versus SDD+C patients. S1 Figure: clinical characteristics in dengue patients. Clinical parameters, including (A) White blood cells, (B) hematocrit, (C) platelet, and (D) NS1, were shown. Data were analyzed using the Kruskal-Wallis test. ∗P < 0.05, ∗∗P < 0.01, and ∗∗∗P < 0.001. S2 Figure: correlation test between clinical characteristics in acute dengue patients. The heat map showed a correlation study between platelet, NS1, WBC, and hematocrit. Significant value marked with a star (∗) inside the area. The blue color indicates a positive correlation, and the red color indicates a negative correlation. WBC: white blood cells; NS1: nonstructural protein 1; OD: optical density. Data were analyzed using the Spearman comparison test. ∗∗P < 0.01. S3 Figure: IL-18 level in healthy, MDF, SDD-C, and SDD+C. The graph showed the difference between groups in IL-18 concentration. Data were analyzed using the Kruskal-Wallis test. ∗∗P < 0.01 and ∗∗∗P < 0.001. [file 7652569.f1.docx]

**Supporting information**

**S1 Table Literature study of increased plasma/serum levels of cytokine/chemokine expression in adult dengue patients**

| **Number** | **Cytokine/Chemokine** | **Reported**  **(Time)** | **References** |
| --- | --- | --- | --- |
| 1 | IL-10 | 21 | [2, 3, 6, 8-16, 21, 25-27, 33, 38-41] |
| 2 | IFN-γ | 19 | [2, 3, 5, 6, 8, 11, 12, 14-16, 24-26, 28, 29, 32-35] |
| 3 | IL-6 | 15 | [2, 3, 5, 9, 11-13, 16, 26, 28, 29, 35, 38-40] |
| 4 | TNF-α | 15 | [1, 2, 6, 8, 13, 15, 16, 22-29] |
| 5 | IL-8 (CXCL-8) | 13 | [1, 6, 8, 12-15, 27, 29, 34, 37-39] |
| 6 | IP-10 (CXCL-10) | 12 | [3, 5, 6, 12, 14, 15, 17, 18, 20, 21, 36, 37] |
| 7 | MCP-1 (CCL-2) | 9 | [3, 5, 6, 13-16, 18, 19] |
| 8 | IL-4 | 7 | [8, 15, 16, 25, 27, 35, 37] |
| 9 | MIP-1β (CCL-4) | 7 | [6, 8, 16-20] |
| 10 | IFN-α | 7 | [2, 5, 6, 15, 21, 26, 30] |
| 11 | RANTES (CCL-5) | 6 | [4, 5, 8, 12, 14, 19] |
| 12 | GM-CSF (CSF-2) | 6 | [5, 6, 15, 16, 25, 35] |
| 13 | IL-15 | 6 | [2, 3, 6, 15, 21, 42] |
| 14 | VEGF (VEGFA) | 4 | [2-5] |
| 15 | IL-13 | 4 | [5, 16, 25, 35] |
| 16 | IL-18 | 4 | [2, 4, 14, 44] |
| 17 | IL-1Rα | 4 | [5, 6, 15, 17] |
| 18 | IL-12 (IL-12p70) | 3 | [2, 12, 29] |
| 19 | MIP-1α (CCL-3) | 3 | [6, 14, 15] |
| 20 | MIG (CXCL-9) | 3 | [12, 14, 21] |
| 21 | IL-17 (IL-17A) | 3 | [5, 41, 43] |
| 22 | IL-1β | 3 | [8, 16, 35] |
| 23 | IL-7 | 2 | [5, 35] |
| 24 | IL-2 | 2 | [16, 25] |

**References**

1. Opasawatchai, A.; Amornsupawat, P.; Jiravejchakul, N.; Chan-In, W.; Spoerk, N. J.; Manopwisedjaroen, K.; Singhasivanon, P.; Yingtaweesak, T.; Suraamornkul, S.; Mongkolsapaya, J.; Sakuntabhai, A.; Matangkasombut, P.; Loison, F., Neutrophil Activation and Early Features of NET Formation Are Associated With Dengue Virus Infection in Human. *Front Immunol* **2018,** 9, 3007.

2. Zimmer, C. L.; Cornillet, M.; Solà-Riera, C.; Cheung, K. W.; Ivarsson, M. A.; Lim, M. Q.; Marquardt, N.; Leo, Y. S.; Lye, D. C.; Klingström, J.; MacAry, P. A.; Ljunggren, H. G.; Rivino, L.; Björkström, N. K., NK cells are activated and primed for skin-homing during acute dengue virus infection in humans. *Nat Commun* **2019,** 10, (1), 3897.

3. Tramontini Gomes de Sousa Cardozo, F.; Baimukanova, G.; Lanteri, M. C.; Keating, S. M.; Moraes Ferreira, F.; Heitman, J.; Pannuti, C. S.; Pati, S.; Romano, C. M.; Cerdeira Sabino, E., Serum from dengue virus-infected patients with and without plasma leakage differentially affects endothelial cells barrier function in vitro. *PLoS One* **2017,** 12, (6), e0178820.

4. Yong, Y. K.; Tan, H. Y.; Jen, S. H.; Shankar, E. M.; Natkunam, S. K.; Sathar, J.; Manikam, R.; Sekaran, S. D., Aberrant monocyte responses predict and characterize dengue virus infection in individuals with severe disease. *J Transl Med* **2017,** 15, (1), 121.

5. Becquart, P.; Wauquier, N.; Nkoghe, D.; Ndjoyi-Mbiguino, A.; Padilla, C.; Souris, M.; Leroy, E. M., Acute dengue virus 2 infection in Gabonese patients is associated with an early innate immune response, including strong interferon alpha production. *BMC Infect Dis* **2010,** 10, 356.

6. Chagan-Yasutan, H.; Ndhlovu, L. C.; Lacuesta, T. L.; Kubo, T.; Leano, P. S.; Niki, T.; Oguma, S.; Morita, K.; Chew, G. M.; Barbour, J. D.; Telan, E. F.; Hirashima, M.; Hattori, T.; Dimaano, E. M., Galectin-9 plasma levels reflect adverse hematological and immunological features in acute dengue virus infection. *J Clin Virol* **2013,** 58, (4), 635-40.

7. Tang, T. H.; Alonso, S.; Ng, L. F.; Thein, T. L.; Pang, V. J.; Leo, Y. S.; Lye, D. C.; Yeo, T. W., Increased Serum Hyaluronic Acid and Heparan Sulfate in Dengue Fever: Association with Plasma Leakage and Disease Severity. *Sci Rep* **2017,** 7, 46191.

8. Cui, L.; Lee, Y. H.; Thein, T. L.; Fang, J.; Pang, J.; Ooi, E. E.; Leo, Y. S.; Ong, C. N.; Tannenbaum, S. R., Serum Metabolomics Reveals Serotonin as a Predictor of Severe Dengue in the Early Phase of Dengue Fever. *PLoS Negl Trop Dis* **2016,** 10, (4), e0004607.

9. Dayarathna, S.; Jeewandara, C.; Gomes, L.; Somathilaka, G.; Jayathilaka, D.; Vimalachandran, V.; Wijewickrama, A.; Narangoda, E.; Idampitiya, D.; Ogg, G. S.; Malavige, G. N., Similarities and differences between the 'cytokine storms' in acute dengue and COVID-19. *Sci Rep* **2020,** 10, (1), 19839.

10. Chen, R. F.; Yang, K. D.; Wang, L.; Liu, J. W.; Chiu, C. C.; Cheng, J. T., Different clinical and laboratory manifestations between dengue haemorrhagic fever and dengue fever with bleeding tendency. *Transactions of the Royal Society of Tropical Medicine and Hygiene* **2007,** 101, (11), 1106-13.

11. Chen, L. C.; Lei, H. Y.; Liu, C. C.; Shiesh, S. C.; Chen, S. H.; Liu, H. S.; Lin, Y. S.; Wang, S. T.; Shyu, H. W.; Yeh, T. M., Correlation of serum levels of macrophage migration inhibitory factor with disease severity and clinical outcome in dengue patients. *Am J Trop Med Hyg* **2006,** 74, (1), 142-7.

12. Patra, G.; Mallik, S.; Saha, B.; Mukhopadhyay, S., Assessment of chemokine and cytokine signatures in patients with dengue infection: A hospital-based study in Kolkata, India. *Acta Trop* **2019,** 190, 73-79.

13. Lin, C. Y.; Kolliopoulos, C.; Huang, C. H.; Tenhunen, J.; Heldin, C. H.; Chen, Y. H.; Heldin, P., High levels of serum hyaluronan is an early predictor of dengue warning signs and perturbs vascular integrity. *EBioMedicine* **2019,** 48, 425-441.

14. Huang, J.; Liang, W.; Chen, S.; Zhu, Y.; Chen, H.; Mok, C. K. P.; Zhou, Y., Serum Cytokine Profiles in Patients with Dengue Fever at the Acute Infection Phase. *Dis Markers* **2018,** 2018, 8403937.

15. Finkelstein, J. L.; Colt, S.; Layden, A. J.; Krisher, J. T.; Stewart-Ibarra, A. M.; Polhemus, M.; Beltrán-Ayala, E.; Tedesco, J. M.; Cárdenas, W. B.; Endy, T.; Mehta, S., Micronutrients, Immunological Parameters, and Dengue Virus Infection in Coastal Ecuador: A Nested Case-Control Study in an Infectious Disease Surveillance Program. *J Infect Dis* **2020,** 221, (1), 91-101.

16. Wang, W. H.; Lin, C. Y.; Chang, K.; Urbina, A. N.; Assavalapsakul, W.; Thitithanyanont, A.; Lu, P. L.; Chen, Y. H.; Wang, S. F., A clinical and epidemiological survey of the largest dengue outbreak in Southern Taiwan in 2015. *Int J Infect Dis* **2019,** 88, 88-99.

17. de-Oliveira-Pinto, L. M.; Gandini, M.; Freitas, L. P.; Siqueira, M. M.; Marinho, C. F.; Setúbal, S.; Kubelka, C. F.; Cruz, O. G.; Oliveira, S. A., Profile of circulating levels of IL-1Ra, CXCL10/IP-10, CCL4/MIP-1β and CCL2/MCP-1 in dengue fever and parvovirosis. *Mem Inst Oswaldo Cruz* **2012,** 107, (1), 48-56.

18. Rathakrishnan, A.; Wang, S. M.; Hu, Y.; Khan, A. M.; Ponnampalavanar, S.; Lum, L. C.; Manikam, R.; Sekaran, S. D., Cytokine expression profile of dengue patients at different phases of illness. *PLoS One* **2012,** 7, (12), e52215.

19. de-Oliveira-Pinto, L. M.; Marinho, C. F.; Povoa, T. F.; de Azeredo, E. L.; de Souza, L. A.; Barbosa, L. D.; Motta-Castro, A. R.; Alves, A. M.; Ávila, C. A.; de Souza, L. J.; da Cunha, R. V.; Damasco, P. V.; Paes, M. V.; Kubelka, C. F., Regulation of inflammatory chemokine receptors on blood T cells associated to the circulating versus liver chemokines in dengue fever. *PLoS One* **2012,** 7, (7), e38527.

20. Becerra, A.; Warke, R. V.; Martin, K.; Xhaja, K.; de Bosch, N.; Rothman, A. L.; Bosch, I., Gene expression profiling of dengue infected human primary cells identifies secreted mediators in vivo. *J Med Virol* **2009,** 81, (8), 1403-11.

21. van de Weg, C. A.; Pannuti, C. S.; de Araújo, E. S.; van den Ham, H. J.; Andeweg, A. C.; Boas, L. S.; Felix, A. C.; Carvalho, K. I.; de Matos, A. M.; Levi, J. E.; Romano, C. M.; Centrone, C. C.; de Lima Rodrigues, C. L.; Luna, E.; van Gorp, E. C.; Osterhaus, A. D.; Martina, B. E.; Kallas, E. G., Microbial translocation is associated with extensive immune activation in dengue virus infected patients with severe disease. *PLoS Negl Trop Dis* **2013,** 7, (5), e2236.

22. Sánchez-Leyva, M.; Sánchez-Zazueta, J. G.; Osuna-Ramos, J. F.; Rendón-Aguilar, H.; Félix-Espinoza, R.; Becerra-Loaiza, D. S.; Sánchez-García, D. C.; Romero-Quintana, J. G.; Castillo Ureta, H.; Velarde-Rodríguez, I.; Velarde-Félix, J. S., Genetic Polymorphisms of Tumor Necrosis Factor Alpha and Susceptibility to Dengue Virus Infection in a Mexican Population. *Viral Immunol* **2017,** 30, (8), 615-621.

23. Senaratne, T.; Carr, J.; Noordeen, F., Elevation in liver enzymes is associated with increased IL-2 and predicts severe outcomes in clinically apparent dengue virus infection. *Cytokine* **2016,** 83, 182-188.

24. Soundravally, R.; Hoti, S. L.; Patil, S. A.; Cleetus, C. C.; Zachariah, B.; Kadhiravan, T.; Narayanan, P.; Kumar, B. A., Association between proinflammatory cytokines and lipid peroxidation in patients with severe dengue disease around defervescence. *Int J Infect Dis* **2014,** 18, 68-72.

25. Maneekan, P.; Leaungwutiwong, P.; Misse, D.; Luplertlop, N., T helper (Th) 1 and Th2 cytokine expression profile in dengue and malaria infection using magnetic bead-based bio-plex assay. *Southeast Asian J Trop Med Public Health* **2013,** 44, (1), 31-6.

26. Tang, Y.; Kou, Z.; Zhang, F.; Yao, X.; Liu, S.; Ma, J.; Zhou, Y.; Zhao, W.; Tang, X.; Jin, X., Both viremia and cytokine levels associate with the lack of severe disease in secondary dengue 1 infection among adult Chinese patients. *PLoS One* **2010,** 5, (12), e15631.

27. Houghton-Triviño, N.; Salgado, D. M.; Rodríguez, J. A.; Bosch, I.; Castellanos, J. E., Levels of soluble ST2 in serum associated with severity of dengue due to tumour necrosis factor alpha stimulation. *J Gen Virol* **2010,** 91, (Pt 3), 697-706.

28. Restrepo, B. N.; Ramirez, R. E.; Arboleda, M.; Alvarez, G.; Ospina, M.; Diaz, F. J., Serum levels of cytokines in two ethnic groups with dengue virus infection. *Am J Trop Med Hyg* **2008,** 79, (5), 673-7.

29. Cruz Hernández, S. I.; Puerta-Guardo, H. N.; Flores Aguilar, H.; González Mateos, S.; López Martinez, I.; Ortiz-Navarrete, V.; Ludert, J. E.; Angel, R. M., Primary dengue virus infections induce differential cytokine production in Mexican patients. *Mem Inst Oswaldo Cruz* **2016,** 111, (3), 161-7.

30. De La Cruz Hernández, S. I.; Puerta-Guardo, H.; Flores-Aguilar, H.; González-Mateos, S.; López-Martinez, I.; Ortiz-Navarrete, V.; Ludert, J. E.; Del Angel, R. M., A strong interferon response correlates with a milder dengue clinical condition. *J Clin Virol* **2014,** 60, (3), 196-9.

31. Oliveira, R. A.; Silva, M. M.; Calzavara-Silva, C. E.; Silva, A. M.; Cordeiro, M. T.; Moura, P. M.; Baptista, P. N. F.; Marques, E. T. J.; Gil, L. H., Primary dengue haemorrhagic fever in patients from northeast of Brazil is associated with high levels of interferon-β during acute phase. *Mem Inst Oswaldo Cruz* **2016,** 111, (6), 378-84.

32. Pal, T.; Dutta, S. K.; Mandal, S.; Saha, B.; Tripathi, A., Differential clinical symptoms among acute phase Indian patients revealed significant association with dengue viral load and serum IFN-gamma level. *J Clin Virol* **2014,** 61, (3), 365-70.

33. Malavige, G. N.; Gomes, L.; Alles, L.; Chang, T.; Salimi, M.; Fernando, S.; Nanayakkara, K. D.; Jayaratne, S.; Ogg, G. S., Serum IL-10 as a marker of severe dengue infection. *BMC Infect Dis* **2013,** 13, 341.

34. Priyadarshini, D.; Gadia, R. R.; Tripathy, A.; Gurukumar, K. R.; Bhagat, A.; Patwardhan, S.; Mokashi, N.; Vaidya, D.; Shah, P. S.; Cecilia, D., Clinical findings and pro-inflammatory cytokines in dengue patients in Western India: a facility-based study. *PLoS One* **2010,** 5, (1), e8709.

35. Bozza, F. A.; Cruz, O. G.; Zagne, S. M.; Azeredo, E. L.; Nogueira, R. M.; Assis, E. F.; Bozza, P. T.; Kubelka, C. F., Multiplex cytokine profile from dengue patients: MIP-1beta and IFN-gamma as predictive factors for severity. *BMC Infect Dis* **2008,** 8, 86.

36. Oliveira, R.; Cordeiro, M. T.; Moura, P.; Baptista Filho, P. N. B.; Braga-Neto, U. M.; Marques, E. T. A. J.; Gil, L., Serum cytokine/chemokine profiles in patients with dengue fever (DF) and dengue hemorrhagic fever (FHD) by using protein array. *J Clin Virol* **2017,** 89, 39-45.

37. Jadhav, M.; Nayak, M.; Kumar, S.; Venkatesh, A.; Patel, S. K.; Kumar, V.; Sharma, S.; Samanta, B.; Deb, S.; Karak, A.; Verma, S.; Talukdar, A.; Kochar, S. K.; Mansukhani, P.; Gandhi, M.; Srivastava, S., Clinical Proteomics and Cytokine Profiling for Dengue Fever Disease Severity Biomarkers. *Omics* **2017,** 21, (11), 665-677.

38. Naranjo-Gómez, J. S.; Castillo, J. A.; Rojas, M.; Restrepo, B. N.; Diaz, F. J.; Velilla, P. A.; Castaño, D., Different phenotypes of non-classical monocytes associated with systemic inflammation, endothelial alteration and hepatic compromise in patients with dengue. *Immunology* **2019,** 156, (2), 147-163.

39. Iani, F. C.; Caldas, S.; Duarte, M. M.; Cury, A. L.; Cecílio, A. B.; Costa, P. A.; Antonelli, L. R.; Gollob, K. J., Dengue Patients with Early Hemorrhagic Manifestations Lose Coordinate Expression of the Anti-Inflammatory Cytokine IL-10 with the Inflammatory Cytokines IL-6 and IL-8. *Am J Trop Med Hyg* **2016,** 95, (1), 193-200.

40. Brasier, A. R.; Garcia, J.; Wiktorowicz, J. E.; Spratt, H. M.; Comach, G.; Ju, H.; Recinos, A., 3rd; Soman, K.; Forshey, B. M.; Halsey, E. S.; Blair, P. J.; Rocha, C.; Bazan, I.; Victor, S. S.; Wu, Z.; Stafford, S.; Watts, D.; Morrison, A. C.; Scott, T. W.; Kochel, T. J., Discovery proteomics and nonparametric modeling pipeline in the development of a candidate biomarker panel for dengue hemorrhagic fever. *Clin Transl Sci* **2012,** 5, (1), 8-20.

41. Fernando, S.; Wijewickrama, A.; Gomes, L.; Punchihewa, C. T.; Madusanka, S. D.; Dissanayake, H.; Jeewandara, C.; Peiris, H.; Ogg, G. S.; Malavige, G. N., Patterns and causes of liver involvement in acute dengue infection. *BMC Infect Dis* **2016,** 16, 319.

42. Azeredo, E. L.; De Oliveira-Pinto, L. M.; Zagne, S. M.; Cerqueira, D. I.; Nogueira, R. M.; Kubelka, C. F., NK cells, displaying early activation, cytotoxicity and adhesion molecules, are associated with mild dengue disease. *Clin Exp Immunol* **2006,** 143, (2), 345-56.

43. Jain, A.; Pandey, N.; Garg, R. K.; Kumar, R., IL-17 level in patients with Dengue virus infection & its association with severity of illness. *J Clin Immunol* **2013,** 33, (3), 613-8.

44. Azeredo, E. L.; Zagne, S. M.; Alvarenga, A. R.; Nogueira, R. M.; Kubelka, C. F.; de Oliveira-Pinto, L. M., Activated peripheral lymphocytes with increased expression of cell adhesion molecules and cytotoxic markers are associated with dengue fever disease. *Mem Inst Oswaldo Cruz* **2006,** 101, (4), 437-49.

45. Vivanco-Cid, H.; Maldonado-Rentería, M. J.; Sánchez-Vargas, L. A.; Izaguirre-Hernández, I. Y.; Hernández-Flores, K. G.; Remes-Ruiz, R., Dynamics of interleukin-21 production during the clinical course of primary and secondary dengue virus infections. *Immunol Lett* **2014,** 161, (1), 89-95.

**S2 Table Clinical characteristics in dengue patients**

| **Variable** | **Acute (n=30)** | **Convalescent**  **(n=30)** | ***P*-value*** |
| --- | --- | --- | --- |
| WBC (mean, 10^6^/L) | 5896.67 | 6738.46 | 0.853 |
| Hematocrit (mean, %) | 38.69 | 37.28 | 0.363 |
| Platelet (mean, 10^9^/L) | 87.17 | 183.58 | **0.002** |
| **Variable** | **Acute MDF (n=10)** | **Acute SDD**  **(n=20)** | ***P*-value*** |
| WBC (mean, 10^6^/L) | 5110 | 6290.00 | 0.061 |
| Hematocrit (mean, %) | 36.68 | 39.28 | 0.053 |
| Platelet (mean, 10^9^/L) | 138.2 | 61.65 | **0.041** |
| **Variable** | **Acute MDF (n=10)** | **Convalescent MDF**  **(n=10)** | ***P*-value^#^** |
| WBC (mean, 10^6^/L) | 5110 | 6200 | 0.1602 |
| Hematocrit (mean, %) | 36.68 | 40.33 | 0.8750 |
| Platelet (mean, 10^9^/L) | 138.2 | 235 | **0.0254** |
| **Variable** | **Acute SDD-C (n=10)** | **Convalescent SDD-C**  **(n=10)** | ***P*-value^#^** |
| WBC (mean, 10^6^/L) | 4380 | 6516.7 | 0.1563 |
| Hematocrit (mean, %) | 41.48 | 37.32 | 0.3125 |
| Platelet (mean, 10^9^/L) | 69.8 | 166.8 | 0.0547 |
| **Variable** | **Acute SDD+C (n=10)** | **Convalescent SDD+C**  **(n=10)** | ***P*-value^#^** |
| WBC (mean, 10^6^/L) | 8200 | 7410 | 0.5566 |
| Hematocrit (mean, %) | 36.80 | 33.56 | 0.5000 |
| Platelet (mean, 10^9^/L) | 53.5 | 136.1 | 0.0547 |

*Data analyzed using the Mann-Whitney test. A significant change was set on the *P*-value < 0.05.

#Data analyzed using the Wilcoxon matched pairs signed rank test. A significant change was set on the *P*-value < 0.05.

**S3 Table Clinical haematological characteristics in dengue patients**

| **Acute** | **MDF**  **(n=10)**  **(a)** | **SDD-C**  **(n=10)**  **(b)** | **SDD+C**  **(n=10)**  **(c)** | ***P*-value**  **(a) vs (b)** | ***P*-value**  **(a) vs (c)** | ***P*-value**  **(b) vs (c)** |
| --- | --- | --- | --- | --- | --- | --- |
| WBC (mean, 10^6^/L) | 5110 | 4380 | 8200 | NS | NS | NS |
| Hematocrit (mean, %) | 36.68 | 41.48 | 36.8 | NS | NS | NS |
| Platelet (mean, 10^9^/L) | 138.2 | 69.8 | 53.5 | **0.046** | **0.014** | NS |

Data analyzed using pairwise correlations of Kruskal-Wallis Test. A significant change was set on the *P*-value < 0.05.

**S4 Table Healthy, acute, and convalescent dengue cytokine/chemokine level**

| **Cytokine/Chemokine** | **Healthy**  **(pg/mL,**  **n = 6) (a)** | **Acute dengue**  **(pg/mL,**  **n = 30) (b)** | ***P*-value**  **(a) vs. (b)** | **Convalescent dengue**  **(pg/mL,**  **n = 30) (c)** | ***P*-value**  **(b) vs. (c)** |
| --- | --- | --- | --- | --- | --- |
| IL-10 | 0.02 | 44.12 | **0.004** | 9.27 | **0.004** |
| IFN-γ | 19.51 | 62.47 | **0.025** | 8.55 | **<0.001** |
| IL-6 | 0.5 | 97.57 | **<0.001** | 19.68 | **<0.001** |
| TNF-α | 32.6 | 40.85 | 0.687 | 12.04 | **0.013** |
| IL-8 (CXCL-8) | 2.05 | 121.64 | **<0.001** | 121.01 | 0.061 |
| IP-10 (CXCL-10) | 157.45 | 15393.77 | 0.06 | 814.03 | 0.018 |
| MCP-1 (CCL-2) | 331.2 | 2866.49 | **0.001** | 665.61 | **<0.001** |
| IL-4 | 0.09 | 0.03 | 0.377 | 0.02 | **0.015** |
| MIP-1β (CCL-4) | 22.96 | 90.64 | 0.076 | 33.41 | **0.025** |
| IFN-α | 19.65 | 171.1 | **0.03** | 1.96 | **<0.001** |
| RANTES (CCL-5) | 3162.18 | 8350.6 | **0.017** | 6522.8 | 0.784 |
| GM-CSF (CSF-2) | ND | 0 | 1 | 0 | 1 |
| IL-15 | 8.27 | 54.23 | **<0.001** | 27.09 | **<0.001** |
| VEGF (VEGFA) | 18.01 | 67.55 | 0.726 | 17.04 | 0.235 |
| IL-13 | 74.98 | 11.64 | 0.083 | 18 | 0.414 |
| IL-18 | 20.6 | 75.28 | **0.015** | 50.47 | 0.086 |
| IL-1Rα | 6.06 | 17.24 | 0.641 | 8.19 | 0.291 |
| IL-12 (IL-12p70) | 1.41 | 1.51 | 0.374 | 1.18 | 0.078 |
| MIP-1α (CCL-3) | 15.9 | 30.7 | 0.19 | 17.1 | 0.069 |
| MIG (CXCL-9) | 1224 | 11427.88 | **0.001** | 9412.9 | 0.428 |
| IL-17 (IL-17A) | 3.98 | 1.17 | **0.007** | 2.66 | **0.019** |
| IL-1β | 14.66 | 5.4 | 0.079 | 10.09 | 0.424 |
| IL-7 | 0.92 | 3.32 | 0.066 | 4.32 | 0.868 |
| IL-2 | 0.8 | 0.5 | 0.561 | 0.34 | 0.403 |

The table represents the mean difference in cytokine concentration in healthy, acute, and convalescent dengue infection. ND, non-detectable. Data analyzed using Mann-Whitney test. A significant change was set on the *P*-value < 0.05.

**S5 Table MDF and SDD cytokine/chemokine concentration in acute and convalescent phase of disease onset**

| **Cytokine/**  **Chemokine** | **Acute MDF**  **(pg/mL,**  **n = 10) (a)** | **Acute SDD**  **(pg/mL,**  **n = 20) (b)** | ***P*-value**  **(a) vs. (b)** | **Convalescent MDF**  **(pg/mL,**  **n = 10) (c)** | **Convalescent SDD**  **(pg/mL,**  **n = 20) (d)** | ***P*-value**  **(c) vs. (d)** |
| --- | --- | --- | --- | --- | --- | --- |
| IL-10 | 15.48 | 59.28 | 0.177 | 0.05 | 13.07 | 0.292 |
| IFN-γ | 110.63 | 38.4 | 0.06 | 6.16 | 9.69 | 0.375 |
| IL-6 | 22.08 | 135.32 | 0.051 | 4.08 | 29.44 | **0.018** |
| TNF-α | 16.41 | 53.06 | 0.094 | 7.07 | 14.66 | **0.039** |
| IL-8 (CXCL-8) | 24.74 | 167.54 | **0.037** | 5.71 | 175.27 | **0.031** |
| IP-10 (CXCL-10) | 1418.5 | 21507.95 | 0.423 | 238.49 | 1101.8 | 0.258 |
| MCP-1 (CCL-2) | 3966.6 | 2287.49 | 0.242 | 408.33 | 808.54 | 0.231 |
| IL-4 | 0.03 | 0.03 | 0.191 | 0.02 | 0.02 | 0.57 |
| MIP-1β (CCL-4) | 51.96 | 110.99 | 0.521 | 17.45 | 41.82 | **0.007** |
| IFN-α | 228.14 | 141.08 | 0.251 | 1.47 | 2.24 | 0.492 |
| RANTES (CCL-5) | 12021.6 | 6515.1 | 0.071 | 6944.3 | 6312.05 | 0.26 |
| GM-CSF (CSF-2) | 0 | 0 | 1 | 0 | 0 | 1 |
| IL-15 | 41.4 | 60.98 | 0.207 | 11.2 | 34.61 | **0.027** |
| VEGF (VEGFA) | 22.18 | 91.43 | 0.891 | 34.23 | 6.3 | 0.304 |
| IL-13 | 9.94 | 12.48 | 0.486 | 9.62 | 22.19 | 0.343 |
| IL-18 | 32.82 | 96.51 | **0.002** | 32.48 | 59.47 | 0.068 |
| IL-1Rα | 5.72 | 23 | 0.209 | 2.7 | 11.07 | **0.045** |
| IL-12 (IL-12p70) | 2.01 | 1.24 | 0.121 | 1.27 | 1.13 | 0.298 |
| MIP-1α (CCL-3) | 19.38 | 36.99 | 0.442 | 14.35 | 18.54 | 0.945 |
| MIG (CXCL-9) | 10266.5 | 12039.14 | 0.982 | 10543.1 | 8818.05 | 0.927 |
| IL-17 (IL-17A) | 1.54 | 0.96 | 0.355 | 2.61 | 2.68 | 0.612 |
| IL-1β | 5.05 | 5.58 | 0.782 | 16.16 | 6.72 | 0.079 |
| IL-7 | 2.6 | 3.64 | 0.869 | 5.27 | 3.84 | 0.895 |
| IL-2 | 0.49 | 0.5 | 0.802 | 0.38 | 0.32 | 0.55 |

The table represents the mean difference in cytokine/chemokine concentration in MDF and SDD patients of acute and convalescent phase of disease onset. Data analyzed using the Mann-Whitney test. A significant change was set on the *P*-value < 0.05.

**S6 Table Cytokine/chemokine concentration in acute SDD-C versus SDD+C patients**

| **Cytokine/Chemokine** | **Acute SDD-C**  **(pg/mL, n = 10)** | **Acute SDD+C**  **(pg/mL, n = 10)** | ***P*-value** |
| --- | --- | --- | --- |
| IL-10 | 21.16875 | 93.16 | 0.211 |
| IFN-γ | 53.119 | 19.99125 | 0.168 |
| IL-6 | 78.42111 | 192.2167 | 0.085 |
| TNF-α | 24.803 | 81.325 | 0.151 |
| IL-8 (CXCL-8) | 84.68 | 259.6011 | 0.462 |
| IP-10 (CXCL-10) | 36504.92 | 2226.126 | 0.266 |
| MCP-1 (CCL-2) | 1926.073 | 2612.772 | 0.369 |
| IL-4 | 0.02 | 0.04125 | 0.441 |
| MIP-1β (CCL-4) | 43.83889 | 171.431 | 0.121 |
| IFN-α | 185.492 | 91.73333 | 0.079 |
| RANTES (CCL-5) | 8183.9 | 4846.292 | 0.384 |
| GM-CSF (CSF-2) | 0 | 0 | 1 |
| IL-15 | 61.185 | 60.75444 | 0.514 |
| VEGF (VEGFA) | 13.98778 | 161.136 | 0.165 |
| IL-13 | 16.281 | 7.7375 | 0.656 |
| IL-18 | 63.994 | 129.025 | **0.006** |
| IL-1Rα | 26.80889 | 18.10429 | 0.634 |
| IL-12 (IL-12p70) | 1.39 | 1.081111 | 0.062 |
| MIP-1α (CCL-3) | 26.71556 | 47.26556 | 0.627 |
| MIG (CXCL-9) | 8572.261 | 15891.22 | 0.165 |
| IL-17 (IL-17A) | 1.225 | 0.7 | 0.515 |
| IL-1β | 7.606 | 3.331111 | 0.1 |
| IL-7 | 2.949 | 4.335 | 0.97 |
| IL-2 | 0.662 | 0.30125 | 0.196 |

The table represents the mean difference in cytokine/chemokine concentration in two groups of severe DENV infection, SDD-C, and SDD+C. Data analyzed using the Mann-Whitney test. A significant change was set on the *P*-value < 0.05.

**S1 Fig. Clinical characteristics in dengue patients.** Clinical parameters, including (A) White blood cells, (B) hematocrit, (C) platelet, and (D) NS1 were showed. Data analyzed using the Kruskal-Wallis test. * *P* < 0.05, ** *P* < 0.01, and *** *P* < 0.001.

**S2 Fig. Correlation test between clinical characteristics in acute dengue patients.** The heatmap showed a correlation study between platelet, NS1, WBC, and hematocrit. Significant value marked with star (_*_) inside the area. The blue color indicates a positive correlation, and the red color indicates a negative correlation. WBC: white blood cells; NS1: nonstructural protein1; OD: optical density. Data analyzed using Spearman comparison test. ** *P* < 0.01.

**S3 Fig. IL-18 level in healthy, MDF, SDD-C, and SDD+C.** The graph showed difference between group in IL-18 concentration. Data analyzed using the Kruskal-Wallis test. ** *P* < 0.01, and *** *P* < 0.001.
